# Study of Hydroquinone Mediated Cytotoxicity and Hypopigmentation Effects from UVB-Irradiated Arbutin and DeoxyArbutin

**DOI:** 10.3390/ijms18050969

**Published:** 2017-05-03

**Authors:** Nai-Fang Chang, Yi-Shyan Chen, Ying-Ju Lin, Ting-Hsuan Tai, An-Ni Chen, Chen-Hsuan Huang, Chih-Chien Lin

**Affiliations:** 1 Department of Cosmetic Science, Providence University, 200, Sec. 7, Taiwan Boulevard, Shalu Dist., Taichung 43301, Taiwan; nfchang@pu.edu.tw (N.-F.C.); yishyan@gm.pu.edu.tw (Y.-S.C.); g1030053@gm.pu.edu.tw (T.-H.T.); g1030068@gm.pu.edu.tw (A.-N.C.); g1040049@gm.pu.edu.tw (C.-H.H.); 2 Department of Medical Research, China Medical University Hospital, 2 Yuh-Der Road, Taichung 40447, Taiwan; yjlin.kath@gmail.com; 3 School of Chinese Medicine, China Medical University, 91 Hsueh-Shih Road, Taichung 40402, Taiwan

**Keywords:** arbutin, deoxyArbutin, hydroquinone, UVB-irradiation, cytotoxicity, hypopigmentation

## Abstract

Arbutin (Arb) and deoxyArbutin (dA) are both effective hypopigmentation agents. However, they are glucoside derivatives of hydroquinone (HQ), which may be decayed into HQ under higher energy environments. Therefore, safety and toxicity are very important issues when considering the usage of these compounds. However, no study has verified the properties of Ultra-Violet B (UVB)-irradiated Arb and dA. In this work, we investigated the cytotoxicity and hypopigmentation effects of UVB-irradiated Arb and dA in Detroit 551 human fibroblast cells and B16-F10 mouse melanoma cells. The results showed that UVB-irradiated Arb and dA have strong cytotoxicity for the fibroblast cells, especially for dA, the caspase-3 is also activated by the treatment of UVB-irradiated dA in Detroit 551 cells. The results correlated with the produced HQ. In addition, UVB-irradiated Arb and dA suppressed the production of melanin in melanoma cells; this is due to the release of HQ that compensates for the UVB triggered Arb and dA decomposition.

## 1. Introduction

Melanogenesis is a part of skin resistance process that may protect skin cells from the damages caused by Ultra-Violet (UV) irradiation [1]. Under sunlight exposure, keratinocytes secrete an important melanogenesis regulator, alpha-melanocyte stimulating hormones (α-MSH), which is cleavage from pro-opiomelanocortin (POMC) and may trigger the microphthalmia-associated transcription factor (MITF) activation through the melanocortin 1 receptor (MC1R) signaling pathway in melanocytes [2,3]. Then, the tyrosinase activity and melanin production are subsequently upregulated in the pigment granules, melanosome. Finally, the produced melanin may be transferred by melanocytes to their attached keratinocytes; the color of skin is also mainly established during the process [4,5]. 

However, from an Asian viewpoint, many people reduce the skin melanin content for beauty purposes; most Asians prefer to brighten skin appearance. Therefore, many skin whitening agents both from natural sources and artificial methods have been investigated by numerous researches [6,7,8,9]. Moreover, a potent hypopigmentation compound, deoxyArbutin (4-[(tetrahydro-2*H*-pyran-2-yl)oxy]phenol, dA), was produced firstly by the team of Raymond E. Boissy [10]. The dA is a tyrosinase inhibitor; it constrains the tyrosine hydroxylase and 3,4-dihydroxyphenylalanine (DOPA) oxidase activities of tyrosinase at a post-translational stage, thereby preventing melanin synthesis in melanocytes. In addition, clinical trials demonstrated that dA is more safe and effective than hydroquinone (HQ) and arbutin (Arb) [11]. 

Nevertheless, Arb and dA are the glucoside derivatives of hydroquinone; our earlier studies have founded that Arb and dA may be decayed into HQ under higher energy conditions, including high temperature storage and UVB treatment condition, especially dA [12,13]. Therefore, safety and toxicity are very important issues when considering the usage of Arb and dA. However, most of the earlier studies regarding to Arb and dA have only determined with their original functions (exclude the effects of released HQ). Although previous study has revealed that dA may suppress tyrosinase activity and melanin synthesis without inducing reactive oxygen species (ROS) or still retain 95% cell viability of the programmed cell death (apoptosis) at the concentrations [14]; but no study has discussed the effects from UVB-irradiated Arb and dA. For this reason, in the presented study, we analyzed the cytotoxicity and hypopigmentation effects in Detroit 551 human fibroblast cells and B16-F10 mouse melanoma cells under the treatments of UVB-irradiated Arb and dA. 

## 2. Results and Discussion

### 2.1. Cytotoxicity Effects of UVB-Irradiated Arbutin and DeoxyArbutin in Detroit 551 Cells

**Figure 1:**
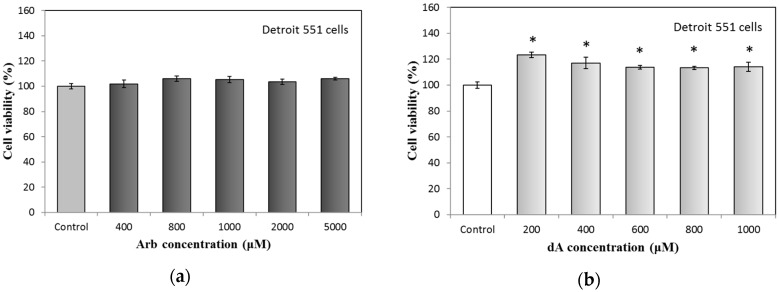
Cytotoxicity test of arbutin and deoxyArbutin on Detroit 551 cells: (**a**) Arbutin; (**b**) deoxyArbutin. Each value represents the mean ± SE (*n* = 3). * *p* < 0.05, compared with the control.

First of all, we analyzed the cell viability of Arb and dA treated Detroit 551 human fibroblast cells using a standard MTT (3-(4,5-dimethylthiazol-2-yl)-2,5-diphenyltetrazolium bromide) method. The results are shown in Figure 1. For Arb, the used concentrations—between 400 to 5000 μM—have no cytotoxic effect on Detroit 551 cells. Similar to the result of Arb in Figure 1a, dA also has no cytotoxicity for Detroit 551 cells if the concentrations are less than 1000 μM. Besides, dA has the function to enhance the growth (up to 13% to 20%) of Detroit 551 cells (Figure 1b). Consequently, we chose 400 μM as a proper concentration of Arb and dA for the following experiments. 

**Figure 2:**
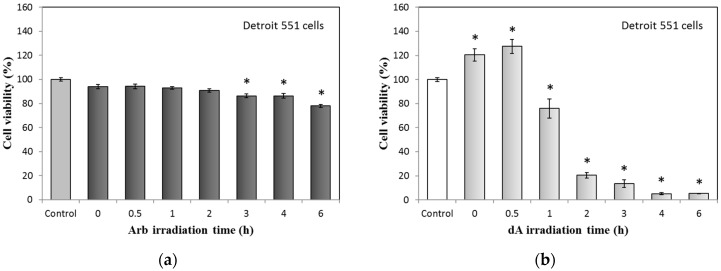
Cytotoxicity test of UVB-irradiated Arb and dA (400 mM) on Detroit 551 cells: (**a**) UVB-irradiated Arb; (**b**) UVB-irradiated dA. Each value represents the mean ± SE (*n* = 3). * *p* < 0.05, compared with the control.

UVB-irradiated Arb and dA also test the effect on cytotoxicity; the results are presented in Figure 2. The cell viabilities of Detroit 551 cells in the treatment of UVB-irradiated Arb (400 μM, irradiation time from 0 to 6 h) gradually decreased. At the 6 h irradiation condition of Arb, cell viability is reduced to around 20% (Figure 2a). However, for the UVB-irradiated dA group, even though the used concentration of dA is the same with Arb (400 μM), cell viabilities of Detroit 551 cells obviously decreased from the conditions of 1 to 6 h irradiation time. At the 2 h irradiation condition of dA, cell viability is reduced to around 20%; for more than 3 h irradiation time, cell viabilities of Detroit 551 cells are reduced to less than 10% (Figure 2b). 

**Figure 3:**
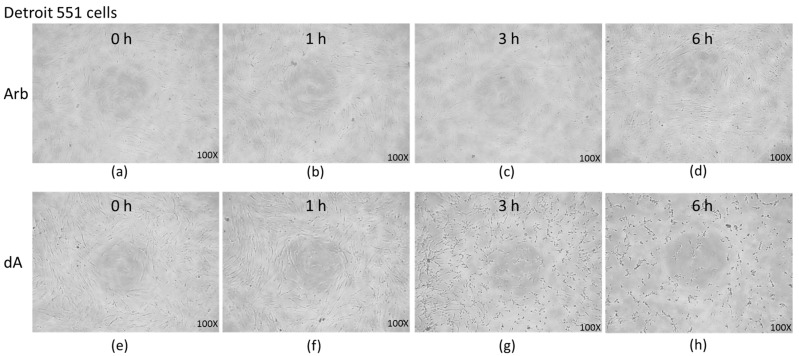
Cell morphology of Detroit 551 cells under the treatment of UVB-irradiated Arb and dA: (**a**–**d**), 0 to 6 h UVB-irradiation of Arb; (**e**–**h**), 0 to 6 h UVB-irradiation of dA.

The cell morphology of Detroit 551 cells under the treatment of UVB-irradiated Arb and dA is shown in Figure 3. From 0 to 6 h UVB-irradiation conditions, the treatment of UVB-irradiated Arb slightly changed the cell pattern at 6 h (Figure 3a–d); few cells are lysed. However, UVB-irradiated dA executes a strong effect on the morphology of Detroit 551 cells from 1 to 6 h conditions. At 3 h and 6 h UVB irradiation conditions, all the Detroit 551 cells are almost ruptured (Figure 3g,h). These results in Figure 3 are consistent with those depicted in Figure 2. 

Our previous studies showed that Arb and dA decomposed into HQ with high-temperature and UV light exposed surroundings [12,13]. Supposedly, the severe cytotoxicity of UVB-irradiated dA to Detroit 551 cells should be provided by the produced HQ. 

### 2.2. Relationships between Hydroquinone and UVB-Irradiated DeoxyArbutin

**Figure 4:**
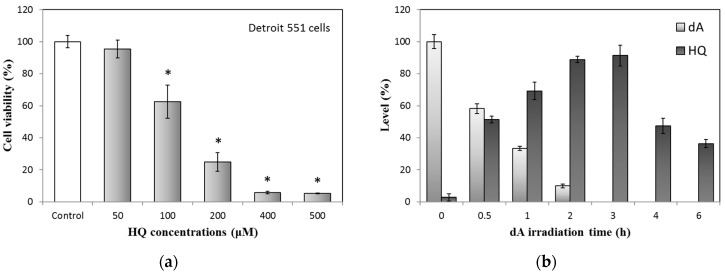
Relationships between HQ and UVB-irradiated dA: (**a**) Cytotoxicity test of HQ on Detroit 551 cells; (**b**) HQ production of UVB-irradiated dA (100 μM). Each value represents the mean ± SE (*n* = 3). * *p* < 0.05, compared with the control.

To confirm the relationships between the released HQ and UVB-irradiated dA, we commenced with the test of cell viability variation of HQ treated Detroit 551 cells; the result is shown in Figure 4a. If the HQ concentrations are higher than 100 μM, Detroit 551 cell viability will reduce to less than 60% of the control. Besides, in Figure 4b, the contents of UVB-irradiated dA and formed HQ were analyzed through the established method, using high performance liquid chromatography (HPLC). The results demonstrated that HQ is quickly amplified with increased irradiation time and at 3 h, this time point has the most abundant HQ level. In contrast, dA reduction is associated with the trend of HQ increase. 

To further compare the results between Figure 2b and Figure 4, we found that the pattern of cell decrease in the UVB-irradiated dA group is similar to that of HQ treatment (Figure 2b and Figure 4a). Moreover, the augmented cytotoxicity of UVB-irradiated dA is also comparable to the accumulated HQ (Figure 2b and Figure 4b). Although the cell viability of UVB-irradiated dA did not totally consist of the concentration of the produced hydroquinone, it might because the original dA has activity to enhance the growth of Detroit 551 cells (Figure 1b). In addition, the cytotoxic effects of HQ to fibroblasts had been estimated by previous study; fibroblast growth of 3 to 5 mM HQ after two days markedly decreased compared with that of control, only 10% to 40% cells stayed alive [15]. Therefore, we can propose again that the severe cytotoxicity of UVB-irradiated dA to fibroblasts is mainly provided by HQ. 

### 2.3. Effects of UVB-Irradiated DeoxyArbutin on Apoptosis Activation in Detroit 551 Cells

Hydroquinone is the main benzene metabolite and a recognized hematotoxic and carcinogenic agent. HQ may cause oxidative stress, DNA damage, cell cycle regulation, apoptosis, and also increased carcinogenic risk [16,17]. Thus, we analyzed whether UVB-irradiated dA may activate the caspase-3 in Detroit 551 cells. 

**Figure 5:**
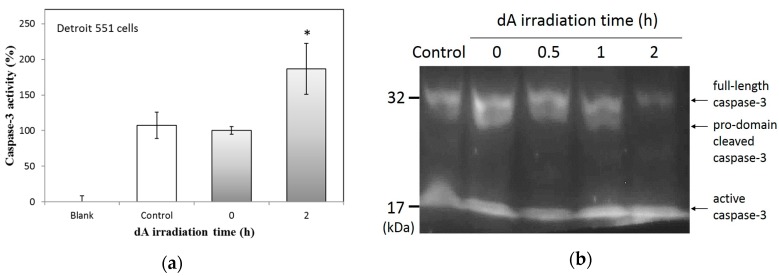
Effects of UVB-irradiated dA on apoptosis activation: (**a**) Caspase-3 activity of Detroit 551 cells under the treatment of UVB-irradiated dA; (**b**) Western bolt analysis of caspase-3 in Detroit 551 cells under the treatment of UVB-irradiated dA. Each value represents the mean ± SE (*n* = 3). * *p* < 0.05, compared with the control.

According to the results in Figure 5a, under the treatment of 400 μM dA, caspase-3 activity is similar to that found in control Detroit 551 cells. In UVB-irradiated dA (2 h) group, the activity of caspase-3 obviously increased to around 190% of the control. To further confirm the activation of caspase-3, we tested the cells using western blot analysis; the results are shown in Figure 5b. The levels of full-length caspase-3 (~32 kDa) reduced with the accumulative UVB irradiation time of dA. The content of pro-domain cleaved caspase-3 (~29 kDa) is also fading in the group of 2 h irradiation time. Moreover, at 1 and 2 h irradiation time groups, the active caspase-3 (~17 kDa) levels are higher. Consequently, UVB-irradiated dA can trigger apoptosis in Detroit 551 cells via the activation of caspase-3. 

### 2.4. Effects of UVB-Irradiated Arbutin and DeoxyArbutin on Melanin Production in B16-F10 Cells

**Figure 6:**
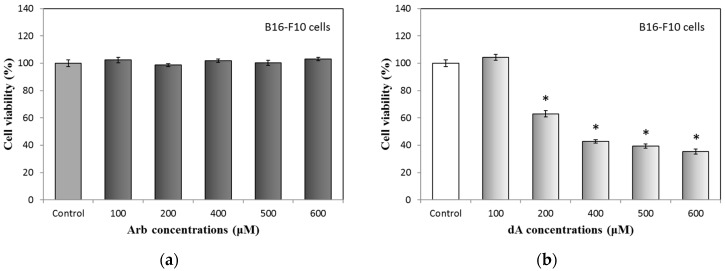
Cytotoxicity test of Arb and dA in B16-F10 cells: (**a**) Arb; (**b**) dA. Each value represents the mean ± SE (*n* = 3). * *p* < 0.05, compared with the control.

Arbutin and deoxyArbutin are both effective hypopigmentation agents, they can reduce melanin synthesis using the anti-tyrosinase activity in melanocytes. Previous study has proven that Arb and dA impede the tyrosinase with a competitive inhibition action [11]. Therefore, we used B16-F10 melanoma cells to check the effects of UVB irradiation for the activities of Arb and dA. We started with a cytotoxicity test of Arb and dA on B16-F10 cells; results are shown in Figure 6. For Arb, fewer than 600 μM Arb treatments, all B16-F10 cells have the same cell viability (Figure 6a). For dA, B16-F10 cells retain around 100% viability only at 100 μM dA condition (Figure 6b). A study from Boissy’s group has revealed that dA is more cytotoxic to human melanocytes than to both human fibroblasts and keratinocytes [18]. This phenomenon is similar to the observations in our study. 

According to these results in Figure 6, we selected the concentration of 100 μM for dA in the subsequent experiment of melanin content assay. In order to compare Arb and dA in the same condition, though the anti-melanogenesis activity of Arb at 100 μM in B16-F10 cells is not significant, we also chose 100 μM as the treatment concentration for Arb. 

**Figure 7:**
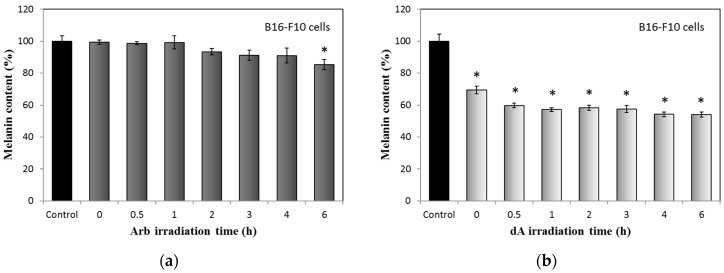
Effects of UVB-irradiated Arb and dA (100 mM) on melanin production in B16-F10 cells: (**a**) UVB-irradiated Arb; (**b**) UVB-irradiated dA. Each value represents the mean ± SE (*n* = 3). * *p* < 0.05, compared with the control.

Effects of UVB-irradiated Arb and dA on melanin production in B16-F10 cells are displayed in Figure 7. In UVB-irradiated Arb group, melanin contents slowly reduced by the increasing irradiation time; the melanin content obviously decreased to about 90% of the control only at the 6 h irradiation time point (Figure 7a). In contrast with Arb, treatments of dA with different UV irradiation times clearly suppressed the melanin production in B16-F10 melanoma cells, including the original dA group (non-irradiated, Figure 7b). 

**Figure 8:**
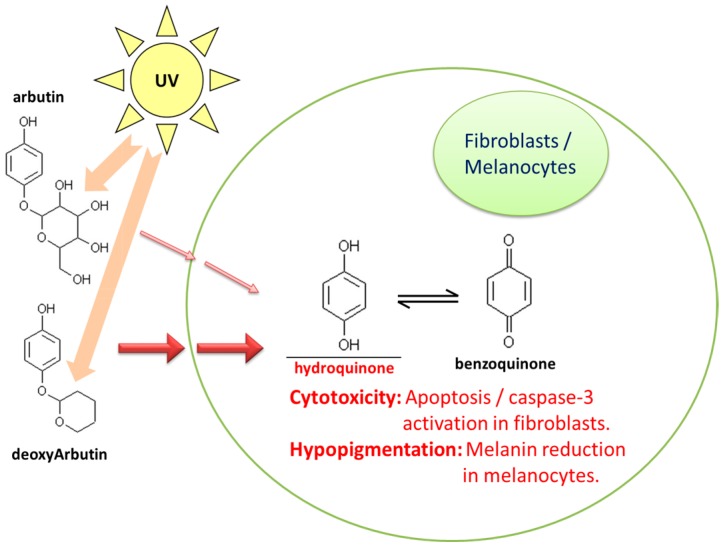
Summary of hydroquinone mediated cytotoxicity and hypopigmentation effects in arbutin and deoxyArbutin under the UVB irradiation.

The used concentrations of Arb and dA in the experiment are the same (100 μM). However, the activities of Arb and dA for the inhibition of melanin production are quite diverse. The function of dA on melanogenesis in B16-F10 mouse melanoma cells is greater than Arb. The result suggests that dA has a less polar structure, tetrahydro-2*H*-pyran, which is different from the glycoside structure of Arb (Figure 8) that can combine to the active site of tyrosinase and also inhibits melanin synthesis effectively in cells [13]. In addition, we observed that melanin contents under the treatments of UVB-irradiated Arb and dA gradually reduced by the increasing irradiation times, even though the original dA group already inhibits more than 30% melanin production, the UVB-irradiated dA group still represses more melanin production than that of non-irradiated dA (Figure 7b). While the HQ concentration is reduced if the UV irradiation times are greater than 3 h (Figure 4b), the molecules decomposed from UVB-irradiated HQ may still influence the production of melanin in B16-F10 cells (Figure 7b). Theoretically, we think that the enhanced hypopigmentation effects of UVB-irradiated Arb and dA are still contributed to by the combining action of Arb or dA and the formed HQ. Moreover, the whole mechanism of UVB-induced decomposition of Arb, dA, and HQ still need to be studied in the future.

## 3. Materials and Methods 

### 3.1. Materials

The deoxyArbutin was obtained from Denjelly Co., Ltd. (Miaoli, Taiwan, R.O.C) and arbutin was purchased from Alfa Aesar (Ward Hill, MA, USA). Hydroquinone was purchased from Wako Pure Chemical Industries (Osaka, Japan). Propylene glycol (PG), sodium hydroxide (NaOH), sodium dodecyl sulfate (SDS), and other chemicals were purchased from Sigma-Aldrich (St. Louis, MO, USA). Dulbecco’s modified Eagle’s medium (DMEM), α-modified essential medium (α-MEM), fetal bovine serum (FBS), trypsin-EDTA, penicillin, and streptomycin were purchased from Gibco BRL/Invitrogen (Carlsbad, CA, USA). The 3-(4,5-dimethylthiazol-2-yl)-2,5-diphenyltetrazolium bromide (MTT) was purchased from USB Affymetrix (Cleveland, OH, USA). The Caspases-3 assay kit with subtracts Asp-Glu-Val-Asp p-nitroanilide (DEVD-pNA) and anti-caspase-3 antibody was purchased from BioVision, Inc. (Milpitas, CA, USA). Deionized distilled water (ddH_2_O) for solutions and buffers was produced by the Milli-Q system (Millipore, Bedford, MA, USA). 

### 3.2. Ultraviolet Irradiation

The 10% PG solutions with 1 × 10^−4^ M Arb or dA were placed in a quartz spectrophotometric tube with 1 ml volume and then irradiated by the UV light source in a light-stability cabinet. The UV lamp was a narrow-band UVB light; the setting wavelength was 312 nm (UVP, Inc., Upland, CA, USA). The lamp light intensity was determined using a radiometer apparatus (UVX Radiometer, UVP, Inc., Upland, CA, USA); the UV intensity was retained at 2.65 mW/cm^2^. The quartz tubes containing solutions of Arb and dA were all protected with aluminum foil before light exposure. All measurements were performed at room temperature. 

### 3.3. Cell Lines, Cell Culture, and Morphology Observation

Detroit 551 (BCRC 60118) human normal fibroblast cells and B16-F10 (BCRC 60031) mouse melanoma cells were all purchased from the Bioresource Collection and Research Center (BCRC, Hsinchu, Taiwan). The Detroit 551 and B16-F10 cells were cultured in α-MEM and DMEM, respectively. Mediums are supplemented with 10% FBS, 2 mM glutamine, 100 mg/mL streptomycin, and 100 U/mL penicillin. Cultured cells were kept in a humidified incubator at 37 °C with 5% CO_2_. The cells were all sub-cultured three to four days each to sustain a regular growth [19]. In the following cell related experiments, the control group is cells treated only with solvent. For morphological analysis, medium in the 96-well plate was removed; the cells were monitored using an inverted microscope (CKX41SF, Olympus, Tokyo, Japan). The images were taken with a digital CCD camera for observation. 

### 3.4. MTT Assay

The cells were planted in 96-well plates with density of 8 × 10^3^ cells/well by the α-MEM (Detroit 551 cells) or DMEM (B16-F10 cells) medium added with 10% FBS. After 24 h, the medium was replaced with new mediums treated with sample at different concentrations for another 24 h. Subsequently, 0.5 mg/mL MTT solution of 100 μL was added to cells and then placed at 37 °C for 30 min. Lastly, after washing with phosphate-buffered saline (PBS), sample cells were lysed using 100 μL DMSO; the absorbance was measured at 540 nm with ELISA microplate reader (BioTek, Seattle, WA, USA). The cell viability was calculated using the percentage of untreated control cells [20]. 

### 3.5. Caspase-3 Activity Assay

Caspase-3 activity in cells was determined by the standard caspase-3 specific substrates with chromophore-labeling, Asp-Glu-Val-Asp p-nitroanilide (DEVD-pNA) [21]. Detroit 551 cells with a density of 6 × 10^5^ cells/well were seeded in 10 cm cell culture dish using α-MEM medium for 24 h and then cells were treated with different samples for next 24 h. After the treatments, collected cells were lysed in 200 μL cold lysis buffer for 20 min. After centrifuge, the prepared cell protein samples were quantified and then analyzed using Asp-Glu-Val-Asp p-nitroanilide specific substrates according to the protocol of the manufacturer (BioVision, Inc.). The spectrophotometric analysis was executed at 405 nm and the caspase activity was expressed as a percentage of the control. 

### 3.6. HPLC Analysis

According to our previous study, we analyzed the contents of deoxyArbutin and hydroquinone using an established HPLC method [12]. Simply, sample from UVB irradiation experiment with 20 μL was injected into the HPLC apparatus (Agilent 1100 series, Santa Clara, CA, USA), separated by a C18 reversed-phase column (Mightysil RP-18, GP 250-4.6, Kanto Chemical, Tokyo, Japan). The UV wavelength was set to 280 nm for the analysis of dA and HQ. The mobile phase consisted of methanol and water with 60:40 (*v*/*v*), pH 7 and the flow rate was 1 mL/min. All used solvents were filtered via a 0.45 μm filter (Millipore). For the assay of HQ production, the percentage of HQ was calculated using the molar ratio of dA to HQ, i.e., 1 g of dA can entirely convert to 0.567 g of HQ. 

### 3.7. Western Blot Analysis

The Detroit 551 cells were cultivated in 6-well plates with the density of 3 × 10^5^ cells/well by α-MEM medium for 24 h. Cells were then cultured for another 24 h in medium supplemented with various testing samples. Then, the cells were washed with PBS buffer and then sonicated with cold lysis buffer (200 μL) for 30 min. In western blot analysis, samples (20 ng protein) were loaded into each well and resolved with 12% SDS-PAGE. Then, the PAGE was electrotransferred onto a membrane of polyvinylidene difluoride (PVDF) using the MiniProtean II apparatus (Bio-Rad Laboratories, Carlsbad, CA, USA). The membrane was incubated with an anti-caspase-3 antibody (primary antibody, BioVision, Inc., Milpitas, CA, USA); then the immune complexes were reacted by the ECL reagents (Millipore, Billerica, MA, USA). The resulting membrane image was collected and analyzed using image analysis software (VIpro Platinum, Version 12.9; UVItec, Cambridge, UK) [22]. 

### 3.8. Melanin Content Assay

For melanin content assay, 8 × 10^4^ cells/well of B16-F10 cells were pre-cultured in 6-well plates in DMEM medium for 24 h and then treated with different samples for another 24 h. The treated cells were washed and then dissolved in 120 μL of 1 N NaOH at 65 °C for 1 h in order to dissolve the melanin. Finally, total melanin amounts in suspensions were analyzed using the ELISA reader at 405 nm absorbance. The melanin content was calculated and presented with percentage of the untreated control [23]. 

### 3.9. Statistical Analysis

The quantitative data were studied using Student’s *t*-tests. Results with three independent experiments are all presented as the mean ± standard error (SE). The calculated *p*-values less than 0.05 are considered as significant. 

## 4. Conclusions 

This work is the first study to investigate the cytotoxicity effect in Detroit 551 human fibroblast cells and the hypopigmentation effect in B16-F10 mouse melanoma cells under the treatments of UVB-irradiated Arb and dA; a summary is presented in Figure 8. 

Our results demonstrated that UVB-irradiated Arb and dA have strong cytotoxicity for the Detroit 551 cells, particularly dA, caspase-3 is activated by UVB-irradiated dA treatment. Moreover, these consequences correlated with the UVB induced HQ formation. In B16-F10 cells, UVB-irradiated Arb and dA suppress the production of melanin; the effect of UVB-irradiated dA on melanin synthesis is also more noticeable than that of UVB-irradiated Arb. The effect gradually improved with the increasing irradiation time associated with HQ, which is contributed by the combining action of Arb or dA and the formed HQ.
